# Who and how to screen for endogenous hypercortisolism in patients with mood disorders

**DOI:** 10.1007/s40618-024-02457-5

**Published:** 2024-11-12

**Authors:** Emanuele Ferrante, Chiara Simeoli, Giovanna Mantovani, Rosario Pivonello

**Affiliations:** 1 Endocrinology Unit, Fondazione IRCCS Ca’ Granda Ospedale Maggiore Policlinico, Milan, Italy; 2 Dipartimento di Medicina Clinica e Chirurgia, Sezione di Endocrinologia, Diabetologia, Andrologia e Nutrizione, Università Federico II di Napoli, Via Sergio Pansini, 5, Naples, 80131 Italy; 3 Department of Clinical Sciences and Community Health, University of Milan, Milan, Italy; 4 Unesco Chair for Health Education and Sustainable Development, University Federico II, Naples, Italy

**Keywords:** Cushing’s syndrome, Non-neoplastic hypercortisolism, Mood disorders, Depression

## Abstract

A strict association exists between mood disorders and endogenous hypercortisolism, namely Cushing’s syndrome (CS). Indeed, CS is characterized by a wide range of mood disorders, such as major depression, generalized anxiety, panic disorders, bipolar disorders up to psychosis, with major depression being the most frequent, with a prevalence of 50–80%, and potentially representing the clinical onset of disease. Despite this observation, the exact prevalence of hypercortisolism in patients with mood disorders is unknown and who/how to screen for endogenous hypercortisolism among patients with mood disorders is still unclear. In this context, an accurate anamnestic and clinical examination are crucial in order to identify those patients who may benefit from CS screening. In particular, the presence of specific signs and symptoms of CS, comorbidities typically associated with CS, and lack of improvement of depressive symptoms with standard treatments can further guide the decision to screen for CS. Anyhow, it is noteworthy that mood disorders represent a cause of functional activation of hypothalamic-pituitary-adrenal (HPA) axis, a condition formerly known as non-neoplastic hypercortisolism (NNH). The differential diagnosis between CS and NNH is challenging. Beyond anamnestic and clinical features, various tests, including measurement of daily urinary cortisol and late-night salivary cortisol, together with low dose-dexamethasone suppression test, are used for initial screening. However, considering their low accuracy, a definitive diagnosis may require a longitudinal follow-up along with second-line dynamic tests like combined dexamethasone-CRH test and desmopressin test. In conclusion, available data suggest the need for a comprehensive assessment and follow-up of individuals with mood disorders to detect possible underlying CS, considering the pitfalls in diagnosis and the overlap of symptoms with other conditions like NNH. Specialized centers with expertise in CS diagnosis and differential testing are recommended for accurate evaluation and management of these patients.

## Introduction

A strict association exists between mood disorders and endogenous hypercortisolism, namely Cushing’s syndrome (CS). However, the exact prevalence of CS in patients with mood disorders is unknown and who/how to screen for CS among patients with mood disorders is still unclear.

Indeed, no systematic studies evaluating hypothalamic-pituitary-adrenal (HPA) axis derangements in patients affected by the large variety of mood disorders are available. However, a review of the currently published data could be useful in suggesting clinical management as well as identifying areas of future research.

Few data are currently available in literature to answer the challenging question “***who to screen for endogenous hypercortisolism among patients with mood disorders***”, however, widening the knowledge on prevalence and characteristics of the most frequently reported mood disorders in CS, as well as performing an accurate anamnestic and clinical examination, could help to identify and better select specific categories of patients in whom the screening of CS might be useful. In particular, the presence of specific signs and symptoms of CS, comorbidities typically associated with CS, and lack of improvement of depressive symptoms with standard treatments can further guide the decision to screen for CS.

The second major challenge, even for expert endocrinologists, is to answer the question “***how to screen for endogenous hypercortisolism among patients with mood disorders***”. In this context, the challenge is represented by the differential diagnosis between CS and mood disorders which are included in non-neoplastic hypercortisolism (NNH). A gold standard test has not been clearly identified, although dynamic evaluation through dexamethasone-CRH (DEX-CRH) test as well as the desmopressin test showed high diagnostic accuracy.

## Methods

Two separate extensive Medline searches were performed in 2023 for the research question by EF and CS. The searches included papers published between 1965 and April 30, 2023 and discrepancies were resolved by discussion. The following search word were included: “Hypercortisolism, Cushing’s Syndrome, Cortisol levels, Depression, Mood disorders, Depressive syndrome, Depressive symptoms, Depressive disorders, Psychiatric symptoms, Psychiatric disorders, Neuropsychiatric disorders, Psychosis, Pseudocushing, non-neoplastic hypercortisolism”.

Search terms were linked to the Medical Subject Headings (MeSH) when possible. Keywords and free words were used simultaneously. Additional articles were identified with manual searches by checking the reference list of relevant review articles, meta-analyses and original articles on this topic.

### Who to screen for endogenous hypercortisolim in mood disorders

The most relevant mood disorders associated with CS are major depression, generalized anxiety, panic and bipolar disorders, up to psychosis [1].

Major depression is the most frequent and severe mood disorder observed in patients with CS with a prevalence of around 50–80% [2–8]. Notably, in up to 25% of patients, major depression has been described as one of the first clinical manifestations at disease onset or earlier [9]. All domains of the depressive syndrome including mood, affective, vegetative, and cognitive functions can be compromised [10], with a spectrum ranging from latent to very severe melancholic forms, up to suicide thoughts and attempts, frequently minimized by patients [1, 2, 11]. The typical manifestation pattern of major depression in CS is characterized by intermittent phases, with frequent and irregular recurrence of exacerbation episodes [10, 12]. A common depressive episode in CS typically lasts 1–2 days, up to no more than 3 days per week, with no regular cyclicity [10], either being present since awakening, remaining through the whole day or the next day, or suddenly occurring during the day, configuring a state known as “mood lability” [12]. During a common depressive episode, a majority of patients reports increased feeling of crying, hopelessness, short spells of sadness, social withdrawal with feelings of discomfort in large groups, an intermittent inability to experience pleasure, rarely reaching persistent anhedonia, and less often self-accusatory or irrational guilt [10, 12]. A minority of CS patients, especially in the first phase of active hypercortisolism, reports “elevated mood” with increased sensitivity to stimuli, “over-reactivity”, increased ambition and restlessness, over-sentimentality, a reduced tolerance even to minor irritations, and easy anger [10, 12]. The most common vegetative manifestations in major depression reported in active CS patients are represented by fatigue, decreased libido, increased or decreased appetite, and sleep disturbances, such as middle, late and early insomnia, alteration in the frequency and type of dreams becoming more bizarre and vivid [1, 10, 12].

Generalized anxiety has been observed in 66% of CS patients [10], whereas panic disorders have been reported in up to 37% of CS patients, especially during the advanced phase of active hypercortisolism [4]. Lastly, bipolar disorders, such as maniac and hypomaniac episodes, have been reported in about 30% of CS patients, possibly representing an early manifestation of CS [2, 3].

Considering that either major depression or bipolar disorders have been described as early clinical manifestations at disease onset or before the onset of the disease [2, 3, 9], all patients with major depression and bipolar disorders might potentially benefit from CS screening. However, the concomitant presence in patients with mood disorders of discriminatory signs of CS, with high specificity, such as proximal myopathy, facial plethora, striae rubrae and easy bruising [13], after the exclusion of glucocorticoid exogenous administration, may strengthen the decision to screen these patients for CS. Similarly, the concomitant presence of comorbidities common in the general population and typically associated with CS, such as metabolic syndrome, visceral obesity, systemic arterial hypertension, dyslipidemia, impaired glucose metabolism up to diabetes mellitus, as well as cardiovascular, thromboembolic and infectious diseases, skeletal damage such as osteopenia or osteoporosis and vertebral fractures [13–16], may further support the decision to screen these patients, especially if these complications are unusual for age and gender [13, 15] (Table 1).

**Table 1:** Discriminatory power of sign and symptoms of Cushing’s syndrome

Sign and symptoms with high specificity (but low sensitivity)	Sign and symptoms with low sensitivity and specificity
Proximal myopathy	Obesity/Weight gain
Facial plethora	Hypertension*
Striae rubrae	Impaired glucose tolerance/Type 2 diabetes mellitus*
Easy bruising	Osteopenia/osteoporosis or vertebral fractures*
	Acne
	Hirsutism
	Menstrual abnormalities
	Decreased libido
	Dorsal/supraclavicular fat pad
	Depression
	Recurrent infections
	Round face
	Atherosclerosis
	Thromboembolic events

*Discriminatory power of these features increases if onset occurs at younger age

Notably, the lack of improvement in mood disorders with antidepressants or anxiolytics drugs, or a peculiar or unexpected resistance to these drugs, may highlight the presence of different unresolved underlying disorders, including CS, which may require further investigation. Indeed, several evidence have shown that depressive symptoms and psychological distress, apparently refractory to standard treatments, in patients screened and confirmed for CS, may improve upon achievement of normal cortisol levels by either surgery, radiotherapy or the use of inhibitors of corticosteroid production [5, 11, 17–23], especially metyrapone in case of CS acute psychiatric states [20]. In a more recent retrospective cross-sectional study performed on 118 patients with CS, depression resolved in 52% of cases after disease remission [24]. Similarly, anxiety disorders may improve after CS remission; indeed, a study described that 6 months after CS treatment, consisting in surgery, irradiation or medical therapy, no patients presented with anxiety disorders [19]. However, because cortisol normalization may require time, support psychotherapy and psychoeducation may be helpful in the meantime, with the addition of psychotropic drugs, especially in severe cases [9]. Furthermore, despite a high normalization rate of depressive symptoms, the use of antidepressant drugs as far as hypnotics is more frequent in remitted patients than in controls, also after long-term follow-up [25].

Noteworthy, considering the challenging clinical condition of the possible presence of cyclic CS, characterized by weeks to months of normal cortisol secretion interspersed with episodes of cortisol excess, the utility of multiple and careful, periodical, long-term screening of CS in patients with mood disorders cannot be underestimated, to avoid missing CS diagnoses [26, 27].

### How to screen hypercortisolism in mood disorders

The second challenge is represented by the differential diagnosis between CS and mood disorders, such as depression, anxiety, and obsessive-compulsive disorders, which are included in NNH. NNH include clinical conditions that stimulate physiologic/non-neoplastic activation of the hypothalamic-pituitary-adrenal (HPA) axis, determining sustained or intermittent hypercortisolism, and sharing with CS several clinical and biochemical features [28]. Apart mood disorders, some additional endocrine and non-endocrine diseases, including polycystic ovary syndrome, obesity, poorly controlled diabetes mellitus, chronic alcoholism, end-stage renal failure, may induce a chronic activation of the HPA axis determining the occurrence of clinical signs of hypercortisolism [28, 29].

Although dynamic biochemical evaluation is crucial for the differential diagnosis between CS and NNH, basal and longitudinal clinical evaluation is also of great significance, in particular in milder cases. In these latter situations, it should be kept in mind that a short-term clinical reassessment, along with patient’s clinical history and duration of symptoms, may guide the diagnosis, since CS tends to worsen over time. Furthermore, the treatment of underlying conditions potentially related to NNH may result in the full recovery of HPA axis function [15, 29].

A retrospective study population compared 32 patients with CS to 23 with NNH [30]: in CS patients ecchymoses, overweight/obesity and osteoporosis were significantly more frequent, while polycystic ovary syndrome was more common in the NNH group. Conversely, the prevalence of many features of CS including muscle weakness, depression, diabetes, hypertension, acne, hirsutism and menstrual disorders were similar in both groups. In a similar study including 60 patients with Cushing’s disease (CD) and 41 patients with NNH, the prevalence of moon face, buffalo hump, easy bruising and erectile dysfunction was significantly higher in patients with CD, whereas acne and headache were more frequent in patients with NNH [31].

In a more recent study, a total of 73 patients (53 classified as CD and 20 as NNH) were prospectively evaluated for a median follow-up period of 56 months [32]. Among NNH patients, obesity, moon face and buffalo hump were the most frequent signs, while diabetes mellitus, hypertension, striae, hirsutism, myopathy, acne and osteoporosis were present in a minority of patients. Interestingly, no NNH patients showed a clinical progression during follow-up. Moreover, in this study, 14 out of 20 NNH patients showed a normalization of cortisol secretion after treatment of those conditions (chronic alcoholism, mood disorders, obesity) determining hyperactivation of HPA axis [32]. Similarly, in another study, a resolution of hypercortisolism was observed in 13 of the 19 patients with NNH after a mean follow-up of 28 months [33]. Therefore, the treatment of the associated conditions, potentially leading to NNH, may resolve the abnormalities of the HPA axis [26, 29].

Regarding biochemical features, HPA axis hyperactivity is a frequent finding in patients with major depression [34], with about 20–30% of patients with major depression showing high cortisol levels [35, 36], and particularly about 50% high evening cortisol levels, indicative of disrupted diurnal cortisol rhythm [34, 37].

Although data are inconsistent and no consensus on the correct differential diagnosis has been reached yet, a series of tests can help to discriminate CS and NNH [28]. The first-line tests in patients with suspected CS are represented by 24 h-urinary free cortisol (UFC), low-dose 1 mg-dexamethasone (DMX) suppression test (DST) and late-night salivary cortisol (LNSC) [13, 26]. A recent meta-analysis summarized the accuracy of different laboratory tests for the diagnosis of CS [38]. Overall, all tests including the above-mentioned ones are characterized by a high sensitivity and specificity (i.e. over 90%). Nevertheless, DST was the most sensitive [98.6%, confidence interval (CI): 96.9-99.4%] and UFC the least sensitive test (94.0%, CI: 91.6-95.7%), while specificity was comparable among different procedures [38]. However, studies involving a group of patients all suffering from a specific disorder were excluded from the analysis, so this study does not specifically address who and how to screen patients with mood disorders.

First-line screening tests (UFC, DST, LNSC) may result abnormal in both CS patients and NNH, such as patients with mood disorders [29]. Particularly, in NNH, false positive results should not be excluded using the measurements of UFC, DST and LNSC levels, because of attenuated sensitivity to the negative glucocorticoids feedback [39]. In addition, different mechanisms involved in derangement of the HPA axis include accentuated post-awakening surge in cortisol, increase in cortisol pulses in the evening and disturbances of circadian rhythm, alteration of CRH secretion and reduction in 5-α-reductase and 11β-hydroxysteroid dehydrogenase type 2 activity [29, 34, 36, 37, 40–43]. UFC levels may be elevated in both conditions; however, in the NNH UFC levels tend to be mildly elevated, remaining almost always within 3-fold of normal concentrations [26]. Focusing on DST, the concomitant use of commonly prescribed psychiatric drugs may impair DMX metabolism, interfering with the interpretation of DST [29]. Specifically, drugs such as carbamazepine and primidone accelerate DMX metabolism by induction of CYP 3A4, therefore reducing the plasma DMX concentrations, and causing false positive results [44–47]. For the same reason the consumption of alcohol should be investigated [13]. Conversely, drugs such as fluoxetine decrease DMX metabolism by inhibition of CYP 3A4, therefore increasing the plasma DMX concentrations, and causing false negative results [13, 47, 48]. However, measuring DMX concomitantly with cortisol, using laboratory-specific ranges of expected values, can reduce the risk for false results [49, 50]. LNSC seems to perform better than UFC and DST in distinguishing CS from NNH, although further studies are needed to confirm these findings [29]. Indeed, most patients with CS lose the normal cortisol diurnal pattern, maintaining persistently elevated cortisol levels in the evening, whereas about 50% of patients suffering from NNH maintain a normal secretion rhythm, albeit on higher settings, with a cortisol nadir at midnight [36, 51]. The few available data on the use of the first-line tests in patients with NNH are summarized in Table 2. Notably, variation in population recruitment, diagnostic criteria of NNH and cut-offs of biochemical tests make the definition of their role even more cumbersome [30–33, 52–58].

**Table 2:** First-line tests (UFC, DST and LNSC) used for the biochemical characterization of NNH patients

Reference	Patients (NNH)	Depressive disorder (*n*)	First-line test	Prevalence of impaired test in whole group (%)	Prevalence of impaired test in depressive disorder (%)
Yanovski et al., 1993	19	Yes (15)	UFC	100	Unk
Martin et al., 2006	3	No	UFC	66.6	NA
Gatta et al., 2007	14	Yes (8)	UFC DST	64 92	Unk
Pecori-Giraldi et al., 2007	23	Yes (Unk)	UFC DST	From 17 to 38	Unk
Reimondo et al., 2008	15	No	UFC DST	6.7 6.7	NA
Valassi et al., 2009	41	Yes (22)	UFC DST LNSC	Unk	Unk
Alwani et al., 2014	20	Yes (6)	UFC DST	40 85	Unk
Arnaldi et al., 2009	26	Yes (15)	UFC DST	Unk	Unk
Tirabassi et al., 2011	18	Yes (12)	UFC DST	Unk	Unk
Moro et al., 2000	30	Yes (7)	UFC DST	100 Unk	Unk
Rollin et al., 2015	56	No	UFC DST	37.5 (total)	NA

UFC: 24 h-urinary free cortisol; DST: 1 mg-dexamethasone suppression testing; LNSC: late-night salivary cortisol

UNK: unknown; NA: not applicable

Conversely, the second-line tests, including corticotrophin-releasing hormone (CRH) test, combined DEX-CRH test and the desmopressin test, seem to be more useful to discriminate between CS, and particularly ACTH-dependent CS, and NNH. As well known, CRH is the most reliable test; although some authors reported an unsatisfactory discriminatory power between CS and NNH [33, 59], the use of a combined ACTH and cortisol criteria showed an excellent diagnostic performance [56].

The DEX-CRH test involves the administration of 2 days of DMX followed by CRH stimulation. The rationale for its use is based on the hypothesis that only patients affected by CS maintain the response of ACTH and cortisol to CRH stimulation after DMX suppression [29, 33]. Although different cut-offs for stimulated ACTH and cortisol have been proposed so far and variable sensitivity and specificity have been reported [60], as a whole this test provides a high diagnostic accuracy. Lastly, desmopressin use relies on the evidence that desmopressin determines a rise in ACTH and cortisol levels only in patients with ACTH-dependent CS, in whom pituitary tumours could aberrantly express vasopressin-receptor type 2. Otherwise, the normal pituitary gland expresses vasopressin-receptor type 3, that presents a low affinity for desmopressin. Data on desmopressin are more limited with respect to DEX-CRH, but also desmopressin has shown a good performance in this setting [60]. Furthermore, considering that CRH is not currently available in many countries which used it for many years, in case of CRH absence the desmopressin test, being less complex and expensive than the DEX-CRH test, may represent a valid alternative [61]. Very recently, a systematic review and meta-analysis compared the diagnostic performance of these tests [62]: pooled sensitivity was 97%, 88% and 84%, while pooled specificity was 92%, 94% and 99% for DEX-CRH, desmopressin and CRH, respectively. Moreover, meta-regression analysis failed to provide a gold standard, but CRH test seems to have a reduced performance compared to the other tests [62]. Sensibility and specificity of second-line tests used in the differential diagnosis between CS and NNH are summarized in Table 3.

**Table 3:** Sensibility and specificity of different second-line tests in the differential diagnosis between CS and NNH in adult patients

Reference	Test	Cut-off	Se (%)	Sp (%)
Yanovski et al., 1993	Dex-CRH	Cortisol at 15′: 38 nmol/L (1.4 µg/dL)	100	100
Martin et al., 2006	Dex-CRH	Cortisol at 15′: 50 nmol/L (1.8 µg/dL)	100	88
Gatta et al., 2007	Dex-CRH	Cortisol at 15′: 110 nmol/L (4 µg/dL) ACTH at 15′: 3.5 pmol/L (16 pg/mL)	100 100	86 85
Erickson et al., 2007	Dex-CRH	Cortisol at 15′: 70 nmol/L (2.5 µg/dL) ACTH at 15′: 5.9 pmol/L (27 pg/mL)	90 95	90 97
Pecori-Giraldi et al., 2007	Dex-CRH	Cortisol at 15′: 38 nmol/L (1.4 µg/dL)	100	62.5
Reimondo et al., 2008	Dex-CRH	Cortisol at 15′: 44 nmol/L (1.6 µg/dL)	93.7	93.3
Valassi et al., 2009	Dex-CRH	Cortisol at 15′: 38 nmol/L (1.4 µg/dL)	86.3	84.7
Alwani et al., 2014	Dex-CRH	Cortisol at 15′: 87 nmol/L (3.2 µg/dL)	94	100
Yanovski et al., 1993	CRH	Sum of post-CRH cortisol levels > 3450 nmol/L (125 µg/dL)	64	100
Arnaldi et al., 2009	CRH	Basal serum cortisol > 331 nmol/L (12 µg/dL) and ACTH peak > 12 pmol/L (54 pg/mL)	91.3	98.2
Tirabassi et al., 2011	CRH	Basal serum cortisol > 331 nmol/L (12 µg/dL) and ACTH peak > 12 pmol/L (54 pg/mL)	96.6	100
Moro et al., 2000	DDAVP	Δ-ACTH ≥ 6 pmol/L 0’ – 30′ (27.2 pg/mL)	86.8	90.7
Pecori-Giraldi et al., 2007	DDAVP	Δ-ACTH ≥ 6 pmol/L 0’ – 30′ (27.2 pg/mL)	81	90
Tirabassi et al., 2010	DDAVP	Δ-ACTH > 6 pmol/L 0’ – 30′ (27.2 pg/mL) Basal serum cortisol > 331 nmol/L (12 µg/dL) and Δ-ACTH > 4 pmol/L (18 pg/mL)	75 90.3	89.8 91.5
Tirabassi et al., 2011	DDAVP	Basal serum cortisol > 331 nmol/L (12 µg/dL) and Δ-ACTH > 4 pmol/L (18 pg/mL)	96.6	100
Rollin et al., 2015	DDAVP	Peak ACTH of 15.8 pmol/L (71.8 pg/mL) Δ-ACTH ≥ 8.1 pmol/L 15′–30′	90.8 88	94.6 96.4

Dex: dexamethasone; CRH: Corticotropin Realising Hormone; DDAVP: desmopressin; CS: Cushing’s Syndrome; NNH: non-neoplastic hypercortisolism

Δ: delta; Se = Sensitivity, Sp = Specificity

## Conclusions

CS is frequently associated with mood disorders, ranging from depression to severe psychosis. However, the prevalence of hypercortisolism in this cohort of patients has not been assessed yet. Furthermore, depression is a well-recognized underlying disease related to NNH, a condition in which functional activation of the HPA axis leads to typical clinical and biochemical signs of CS. The differential diagnosis between CS and NNH is challenging, and a gold standard test has not been clearly identified, although dynamic evaluation through DEX-CRH test as well as desmopressin test showed high diagnostic accuracy. In this context, both clinical and biochemical follow-up are crucial to achieve a correct diagnosis. Patients with mood disorders and clinical suspicion of hypercortisolism, despite the likelihood of a low pre-test probability of endogenous CS, should be referred to centers with expertise in the differential diagnosis of CS, where appropriate anamnestic, clinical and biochemical screening, a longitudinal follow-up and, if necessary, second-line dynamic tests can be performed.
